# Lemierre’s syndrome with isolated external jugular vein thrombosis caused by *Streptococcus intermedius*

**DOI:** 10.1016/j.idcr.2022.e01495

**Published:** 2022-04-04

**Authors:** Rioto Suzuki, Mari Terayama, Minoru Tanda, Gaku Takahashi

**Affiliations:** Advanced Critical Care and Emergency Center, Iwate Medical University Hospital, Japan

**Keywords:** Lemierre’s syndrome, *Streptococcus intermedius*, Septic thrombophlebitis, Rheumatoid arthritis

## Abstract

An 85-year-old woman with a history of rheumatoid arthritis fell due to unsteadiness and visited our emergency room due to head injury. The patient had minor head trauma and lip and oral cavity injuries, and she presented with fever. Laboratory tests showed high inflammatory marker levels. Moreover, there were indicative of urinary tract infection. Thus, the patient was admitted to our hospital. Blood cultures performed upon admission revealed the presence of *Streptococcus intermedius*, and contrast-enhanced computed tomography scan showed solitary right external jugular vein thrombosis and multiple abscesses in both lungs. Hence, the patient was diagnosed with Lemierre’s syndrome, and antimicrobial agents and anticoagulants were administered. The patient developed left pleurisy due to inflammation caused by lung abscesses, which improved with thoracic drainage. Her condition improved satisfactorily, and she was then discharged. There are only few studies about Lemierre’s syndrome caused by *S. intermedius* and even lesser cases involving external jugular vein thrombosis. Herein, we report a relatively rare case of Lemierre’s syndrome with isolated external jugular vein thrombosis.

## Introduction

Lemierre’s syndrome was first described by Dr. Andrè Lemierre in 1936 [1]. It is an oropharyngeal infection with septic thrombophlebitis of the internal jugular vein caused mainly by *Fusobacterium necrophorum*, an anaerobic gram-negative rod. Due to the septic thrombophlebitis, the patient presents septic embolism mainly affecting the lungs and possibly other organs [1], [2]. Herein, we present a case of Lemierre’s syndrome with isolated external jugular vein thrombosis caused by *Streptococcus intermedius*, which is a rare causative agent.

## Case report

An 85-year-old woman was treated with baricitinib 2 mg/day for rheumatoid arthritis based on the discretion of her family physician. The patient fell about a week prior to her hospital visit and sustained lip and oral cavity injuries. Then, she fell again due to unsteadiness. Moreover, she was bruised at the back of her head and was bleeding. Hence, on the same day, she visited the emergency room. Upon consultation, there was a 2-cm laceration at the back of the head. The wound was cleaned and sutured with a stapler.

During examination, she was conscious and alert. Her vital signs were as follows: heart rate, 115 beats/min; blood pressure, 127/87 mmHg; and body temperature, 37.8-°C. Her blood oxygen saturation level at room air was 96%. Chest auscultation revealed no rales. Further, there were no findings indicative of pharyngitis and tonsilitis, and she did not complain of any subjective symptoms.

Table 1 shows the laboratory data upon admission. The patient’s white blood cell count in the peripheral blood, predominantly that of neutrophils, increased. Her C-reactive protein, transaminase, and D-dimer levels were also high. Although a small number of white blood cells were found in the urine qualitative test, the urinalysis results were normal.

**Table 1:** Laboratory findings upon admission.

Hematology		Biochemistry and serology		Coagulation	
WBC (/μL)	13,660	TP (g/dL)	6.6	PT (sec)	14.1
Neut (%)	85.9	Alb (g/dL)	3.1	PT-INR	1.16
Lym (%)	6.4	T-bil (mg/dL)	0.4	APTT (sec)	31.8
Mo (%)	4.7	AST (U/L)	45	Fbg (mg/dL)	511
Eos (%)	0.4	ALT (U/L)	41	D-D (μg/mL)	18.7
Baso (%)	0.0	γ-GTP (U/L)	63		
RBC (×10^6^/μL)	306	LDH (U/L)	522	Urinalysis	
Hb (g/dL)	9.0	CK (U/L)	357	Protein (g/gCr)	0.30
Ht (%)	27.2	BUN (mg/dL)	22.0	Glucose	–
Plt (×10^4^/μL)	27.1	Cre (mg/dL)	1.82	RBC (/HPF)	< 1
		Na (mmol/L)	135	WBC (/HPF)	< 1
		K (mmol/L)	4.6	Hyalyne cast	+
		Cl (mmol/L)	98		
		Ca (mg/dL)	8.7		
		CRP (mg/dL)	25.14		

WBC: white blood cell, Neut: neutrophil, Lym: lymphocyte, Mo: monocyte, Eos: eosino-phil, Baso: basophil, RBC: red blood cell, Hb: hemoglobin, Ht: hematocrit, Plt: platelet, TP: total protein, Alb: albumin, T-bil: total birilubin, AST: aspartate aminotransferase, ALT: alanine aminotransferase, γ-GTP: gamma-glutamyl transpeptitase, LDH: lactate dehydrogenase, CK: creatinine kinase, BUN: blood urea nitrogen, Cre: creatinine, CRP: C-reactive protein, PT: prothrombin time, PT-INR: prothrombin time-international nor-malized ratio, APTT: activated partial thromboplastin time, Fbg: fibrinogen, D-D: d-dimer, HPF: high power field

**Fig. 1:**
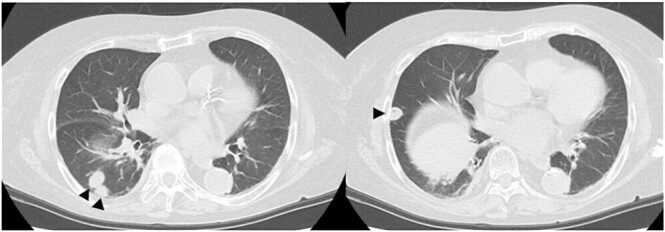
Non-contrast-enhanced computed tomography scan showed multiple nodular shadows in the right lung upon admission (black arrow heads).

Fig. 1 depicts the imaging findings upon admission. Non-contrast-enhanced computed tomography (CT) scan showed multiple nodular shadows in the right lung. Thus, septic pulmonary embolism, metastatic lung tumor, and pulmonary cryptococcosis were considered.

**Fig. 2:**
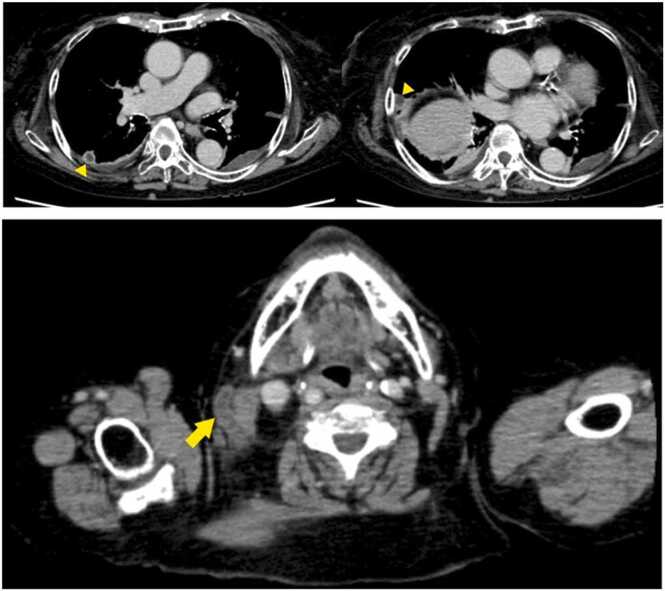
(A) Contrast-enhanced computed tomography scan on day 6 of admission revealed a low-density area inside the multiple lung nodule shadows, and a diagnosis of multiple lung abscesses was made (yellow arrow heads). (B) There was isolated right external jugular vein thrombosis (yellow arrow). (For interpretation of the references to colour in this figure, the reader is referred to the web version of this article.)

After admission, treatment with ceftriaxone 2 g/day for suspected urinary tract infection was started. In addition, continuous intravenous heparin (5000 IU/day) was provided to prevent deep vein thrombosis. Blood culture was performed upon admission, and results, which were obtained on day 3 of admission, showed gram-positive cocci in chain. In addition, multiplex polymerase chain reaction revealed the presence of *Streptococcus spp*. The urine culture result was negative; thus, urinary tract infection was ruled out. Transthoracic echocardiography was performed by a cardiologist to assess for infective endocarditis, and the absence of verrucae was confirmed. On day 5 of admission, the presence of S. anginosus was identified in the blood culture, and added treatment with clindamycin 1200 mg/day was then started. Contrast-enhanced CT scan of the whole body was performed on day 6 of admission, and abscess formation was highly considered. The multiple nodular shadows observed in the right lung on CT scan upon admission decreased. Hence, lung abscesses caused by septic embolism were suspected due to the low-density area inside and the presence of left pleural effusion and passive atelectasis. Isolated right external jugular vein thrombosis was also identified (Fig. 2). Nevertheless, there was no abscess formation in other organs. The final blood culture result revealed *S. intermedius*. Hence, the patient was diagnosed with Lemierre’s syndrome based on the imaging findings. Then, she was examined by a dentist who confirmed the presence of lip and oral cavity injuries sustained after falling. However, periodontitis and caries were not observed. Continuous intravenous heparin at a dose of 5000–10,000 IU/day with a target activated partial thromboplastin time ratio of 1.5–2.5 for thrombophlebitis was continually administered. On day 12 of admission, the patient experienced respiratory failure due to worsening of left pleural effusion and inflammation. We believed that inflammation had spilled over from the lung abscess to cause pleurisy. Thus, thoracic drainage was performed, and the antimicrobial agent was modified to tazobactam/piperacillin 9 g/day. In the case, pleural effusion was an exudative effusion with neutrophil predominance (91.1–%), with the findings of pneumonia-associated pleural effusion (Table 2). Bacterial culture of the pleural effusion had negative results. Inflammation improved immediately after starting thoracic drainage, and the procedure was completed on day 16 of admission. The antimicrobial agent was changed to amoxicillin/clavulanic acid 2000 mg/day on day 22 of admission, and anticoagulation therapy was modified to edoxaban tosilate hydrate 30 mg/day on day 23 of admission. Contrast-enhanced CT scan was performed on day 30 of admission, and the disappearance of right external jugular vein thrombosis and multiple lung abscesses was confirmed. Antimicrobial and anticoagulation therapies were discontinued on day 37 of admission, and the patient was discharged to home on day 38. The patient visited our hospital on day 42. There was no recurrence of infection, and the treatment was completed.

**Table 2:** Pleural effusion test results on day 11 of admission.

Hematology		Biochemistry and serology		Culutre	
WBC (/μL)	9810	TP (g/dL)	3.9	Bacteria	–
Neut (%)	91.0	Alb (g/dL)	1.7	Mycobacterium	–
Lym (%)	5.0	LDH (U/L)	360		
Mo (%)	1.0	T-col (mg/dL)	82		
Eos (%)	1.0	TG (mg/dL)	30		
Baso (%)	0.0	AMY (U/L)	21		
RBC (×10^6^/μL)	0.02	Glu (mg/dL)	149		
Hb (g/dL)	0.2	pH	7.7		
		ADA (IU/L)	25.3		

WBC: white blood cell, Neut: neutrophil, Lym: lymphocyte, Mo: monocyte, Eos: eosino-phil, Baso: basophil, RBC: red blood cell, Hb: hemoglobin, TP: total protein, Alb: albumin, LDH: lactate dehydrogenase, T-col: total cholesterol, TG: triglyceride, AMY: amylase, Glu: glucose, pH: potensial hydrogen, ADA: adenosine deaminase

## Discussion

Based on a previous research, the incidence of Lemierre’s syndrome was one case per million people per year [3]. However, several studies showed that the incidence has been increasing in recent years. Data from Denmark revealed that the incidence increased to 3.6 cases per million people per year [4]. In the days when antimicrobials were not used, the mortality rates of Lemierre’s syndrome, embolism, and endocarditis were 32–90%, 25%, and 12.5%, respectively. This condition remains a potentially life-threatening syndrome. However, recent studies showed that is mortality rate decreased to 0–18% [3], [4], [5], [6]. Moreover, it is caused by anaerobic bacteria, primarily *F. necrophorum*, which are members of the normal flora in the middle pharynx. However, *S. intermedius* is a rare causative agent of Lemierre’s syndrome, with only a few cases reported in the literature [6], [7], [8], [9], [10], [11]. Moreover, it is a gram-positive, microaerophilic coccus, which is a normal flora in the oral cavity and respiratory and gastrointestinal tracts. It is a viridans Streptococcus, and it belongs to the anginosus group, formerly known as the *S. milleri* group, along with *S. anginosus* and *S. constellatus*. These organisms are unique among the viridans streptococci because they are pyogenic. *S. intermedius* is the most virulent among them and is the most likely cause of abscess formation. Abscesses can develop in the liver, brain, skin, and heart valves even in immunocompetent patients [12].

Lemierre’s syndrome causes thrombophlebitis of the internal veins. However, in the current case, the patient presented with isolated right external jugular vein thrombosis. Thrombophlebitis caused by bloodstream infection is less likely to occur in the external jugular vein than in the internal jugular vein because the former is not usually supplied by the veins of the oral cavity and tonsils. Suzuki et al. [13] reported a case of Lemierre’s syndrome with isolated external jugular vein thrombosis, and a variant in which the veins of the oral cavity and tonsils [14] are connected to the external jugular vein, which may be involved in isolated external jugular vein thrombosis.

In this case, treatment was started upon admission based on the initial diagnosis of urinary tract infection. However, the urine culture result was negative, and urinary tract infection was ruled out. Thus, abscess formation in the organs was suspected based on the blood culture results, and the patient was eventually diagnosed with Lemierre’s syndrome based on contrast-enhanced CT scan findings. However, there was no common pharyngeal infection that could cause Lemierre’s syndrome, and oral infection was ruled out by the dentist. Therefore, oral cavity injury from the fall is the only possible cause of bacteremia. The immunosuppressive effect of baricitinib, which is used for the management of rheumatoid arthritis, may play a role in the development of Lemierre’s syndrome in patients without evident pharyngeal or oral infections. Nevertheless, this notion has not been confirmed to date, and further accumulation of cases is required. In this study, contrast-enhanced CT scan showed no evidence of abscess in any organs other than the lungs, and infective endocarditis was not identified on transthoracic echocardiography. During the disease course, pleurisy developed, and thoracic drainage was required. Thereafter, the condition of the patient improved progressively.

The need for anticoagulation therapy in Lemierre’s syndrome has been debated. Despite indications for the development of new thromboembolic complications several patients with this condition do not receive anticoagulation agents due to the high risk of bleeding [15]. In the current patient, anticoagulation treatment was continued, and it was modified from continuous intravenous heparin to direct oral anticoagulant. Nonetheless, bleeding complications did not occur. In an observational study conducted in Sweden, most patients with Lemierre’s syndrome recovered well without anticoagulation treatment [16]. However, more cases should be assessed in the future.

Herein, we report a case of Lemierre’s syndrome caused by *Streptococcus intermedius*, a rare causative organism, and isolated external jugular vein thrombosis. Although Lemierre’s syndrome is a forgotten disease, its incidence has been increasing in recent years, and it can be fatal. Hence, this condition should be considered as a differential diagnosis even in atypical cases, as in this one. Further, early diagnosis and therapeutic intervention are essential.

## CRediT authorship contribution statement

(1) The conception and design of the study, or acquisition of data, or analysis and interpretation of data, (2) drafting the article or revising it critically for important intellectual content, (3) final approval of the version to be submitted, All authors meet the above ICMJE authorship criteria.

## Consent

Written informed consent was obtained from the patient for publication of this case report and accompanying images. A copy of the written consent is available for review by the Editor-in-Chief of this journal on request.

## Conflict of interest

The authors state that they have no Conflict of Interest.
